# Communication Skills (CS) training of physicians in China and its role in actual challenges of patient-physician relationship: a cross-sectional survey

**DOI:** 10.1186/s12909-022-03830-9

**Published:** 2022-11-12

**Authors:** Junfeng Du, Gwendolyn Mayer, Elisabetta Posenato, Svenja Hummel, Ali Zafar, Till Bärnighausen, Jobst-Hendrik Schultz

**Affiliations:** 1 Department of Plastic Surgery, Liyuan Hospital, Tongji Medical College, Huazhong University of Science and Technology, Wuhan, China; 2 Department of General Internal Medicine and Psychosomatics, Heidelberg University Hospital, Heidelberg, Germany; 3 Heidelberg Institute of Global Health, Heidelberg University Hospital, Heidelberg, Germany

**Keywords:** Medical education, Communication skills, Doctor-patient-relationship, China, Patient-centered communication

## Abstract

**Background:**

The Chinese healthcare system is affected by frequent disputes between physicians and patients. Although recent reforms have contributed towards improving the patient-physician relationship, distrust in physicians is still high. Communication skills (CS) training of physicians holds the key to improving patient confidence and diffusing stressful situations. This survey reports on the status of CS training in medical education in China, and the experiences and attitudes of physicians towards CS training.

**Methods:**

A cross-sectional survey was conducted at medical institutions across China. A questionnaire developed for this study included the status of CS training, current aspects of patient-physician relationships, perceived own CS and patient-centeredness with Likert-scaled items from 1 (most negative) to 6 (most positive). Physicians’ attitude towards CS training was measured with the Communication Skills Attitude Scale (CSAS) and its subscales PAS (Positive Attitude Scale) and NAS (Negative Attitude Scale). Data were analyzed descriptively and for group differences between the hospital level and operating vs. non-operating physicians. Binary logistic regression analysis was done to find associations explaining the occurrence of verbal and physical attacks and the role of CS attitudes.

**Results:**

Out of 1080 questionnaires, 772 physicians met inclusion criteria. A total of 466/772 participants (60.4%) had received at least one CS training during their career. The participants rated the current situation related to patient-physician relationship in China as highly stressful (mean = 4.52, SD = 1.26, 95% CI: 4.43–4.60), experiencing verbal attacks in the past three years once a year in 372/772 cases (48.2%) and physical attacks 111/772 times (14.4%). The mean PAS was 62.96 (SD = 7.63, 95% CI: 62.41–63.47). Being female was associated with increased risk of verbal attacks (OR = 1.51, 95% CI: 1.01–2.25) while working in a tertiary hospital and showing high levels of PAS decreased this risk (OR = 0.62, 95% CI: 0.43–0.89, and OR = 0.95, 95% CI: 0.93–0.98). Having received a previous CS training decreased the odds of physical attacks (OR = 0.54, 95% CI: 0.35–0.83).

**Conclusions:**

A majority of Chinese physicians showed a high positive attitude towards CS training, were trained in CS and would value further training. Our results highlight that CS training is likely to promote patient-centered communication and reduce attacks against physicians. Both of these effects are to improve the patient-physician-relationship in the long run. More CS training should be offered to Chinese physicians, especially at secondary- and primary-care hospitals, where such practices remain infrequent.

**Supplementary Information:**

The online version contains supplementary material available at 10.1186/s12909-022-03830-9.

## Introduction

Healthcare systems around the world have evolved significantly in recent decades, challenging the traditional understanding of patient-physician relationships. China’s healthcare industry has also witnessed significant developments, especially in the past two decades with rapid improvements in medical services and public health [1, 2]. The 2009 launched medical reform aimed at universal health coverage and access to basic healthcare for all citizens by 2020; main strategies included the establishment of hospital management systems, drug supply systems, improved supervision and security, telemedicine for rural areas and more [1]. Health insurance coverage was achieved as early as 2011 for 95% of 1.3 billion inhabitants for basic services [3]. However, despite many efforts and investments in the healthcare system, stressful encounters between physicians and patients or their attendants remain a challenge for medical professionals in China [4]. In 2002, about 98% of hospitals had experienced medical disputes and 70% of them were based on patient complaints related to inadequate communication with physicians [5]. Several studies have pointed out the difficulties physicians face in China while dealing with patients [6, 7] and the ever-increasing patient distrust in healthcare professionals [8–10]. Hospitals report a high prevalence of verbal abuse, psychological violence [11] and increasing violent attacks against hospital staff [7, 12, 13]. This difficult patient-physician relationship is indicated as the top stressor for Chinese physicians and has been associated with burnouts [14].

Causes for the tense patient-physician relationship have been identified in the relatively poor quality of life of Chinese physicians due to high workload and low salaries [15]. Especially primary health care doctors have received low levels of training, leading young physicians to leave their profession [16]. Finally, the social image and professional reputation of physicians suffers from ongoing patient-physician conflicts [17]. Studies have also observed a declining interest in studying medicine among young people and the latest medical education reforms have set ambitious goals in enabling health professionals [18]. The majority of contemporary medical schools in China offer a five-year-program, leading to a bachelor’s degree. By 2013, China set the so-called "5 + 3" rule that includes three years of residency training after the bachelor’s degree [19]. Physicians from the preceding generation were educated under the Soviet model and by 2014 more than half of the Chinese doctors had received vocational diplomas, that required only three years of study.

Patient-centered communication is considered important for effective patient-physician communication and better clinical outcomes [20–22]. In recent years, many high-income, Western countries have developed national education frameworks for ensuring teaching of communication skills (CS) as a major competency for physicians [4, 23, 24]. China adopted the Western model of teaching CS to physicians and medical students about 15 years ago [25]. Huge efforts were put in implementing and testing the efficacy of CS training in a variety of settings with several target groups (students, interns, physicians, specialists and nurses). Training included a broad range of competencies such as structuring a talk, providing knowledge, eliciting patient's perspectives, and breaking bad news [25]. A recent survey revealed that a major share of medical colleges that offered a 5 to 8 year educational program provided training with simulated patients, often recruited from among medical students or staff [26]. Studies reporting on the details of the clinical scenarios referred to settings such as history taking, communicating with the family, or empathy building [27]. Although the efforts to teach CS and establish patient-centered communication have shown promising results [8], this approach is not common practice in Chinese clinical settings [28, 29]. Tertiary-care hospitals seem to be faring better in clinical aspects [30, 31] but a national framework for medical professionalism among Chinese physicians is yet to be enforced [10].

While current literature provides promising insights into the Chinese efforts to teach CS to physicians and establish patient-centered communication at hospitals, studies have either focused on specific roles or professions (such as patients, students or a single medical specialization) [25] or they are restricted to a few locations [32–34].

### Aims and objectives

In this study, we provide an up-to-date, China-wide overview of physicians’ experiences and attitudes towards CS training; how physicians relate to it and its clinical consequences, highlighting how CS can affect physicians in their work with patients. The primary aim of the survey was to determine the status and prevalence of CS training among practicing medical physicians in China. The study further investigated the current aspects of patient-physician relationship, perceived own CS and patient-centeredness, and attitudes towards CS in general. We intended to analyze potential associations of factors contributing to patient-physician disputes.

## Methods

### Study design

A cross-sectional survey was conducted between July 2020 and October 2020. An online questionnaire was developed for the purpose of the study and distributed in China via professional contacts at medical institutions. We started distributing the survey at the Tongji Medical College in Wuhan. Participants then redistributed the link further and to clinical institutions all over China. Only graduate or resident physicians practicing at any medical institution in China were eligible to participate in the survey after giving their consent to data privacy regulations and use of data for research purposes. Exclusion criteria were not being a medical doctor e.g. nurses or paramedical staff. Participation was voluntary and the consent to participate was included within the questionnaire. Ethical approval for the study was given by the Ethics Commission of the Medical Faculty of Heidelberg (S-145/2020).

### Measurements

An interdisciplinary research group at the Heidelberg University Hospital including physicians, medical communication experts and psychologists developed the questionnaire based on previously validated instruments. The questionnaire was then translated into the Chinese language with the help of two native Mandarin speakers with appropriate English-language skills.

The questionnaire consisted of four sections: The first part included items regarding sociodemographic data (age, gender, education, workplace, specialty, hospital level, years of experience) and a self-assessment of own communication skills. The second part consisted of questions on the patient-physician relationship situation in China and asked for the relevance of CS for clinical work, the quality of the current patient-physician relationship in general, and a rating how stressful the relationship was felt. All items were asked for with a 6-point Likert scale while 1 was the most negative and 6 the most positive option to be chosen. Participants were then asked, how often they had experienced a verbal or physical attack in the past three years by a patient or his/her family with options from 0 (never), 1 (once a year), 2 (once a month), 3 (once a week), and 4 (once a day). Then the participants were asked about the best way to resolve disputes and potential causing factors of the current patient-physician relationship, such as low communication skills, lack of time, or the new medical policy that was initiated by the health reforms of the recent years. Subsequently, the use of specific patient-centered communication examples was rated, i.e. to inform patients about advantages and disadvantages of a treatment, to take patients’ opinion into account when choosing a treatment plan, and to accept the decision of a patient even if it is not the own favorite. Moreover, the participants rated if good patient-physician relationship had a positive effect on clinical outcomes. The third part asked about teaching of patient-physician CS. This section started with the question, if CS could be taught. Then, the participants were asked, if they had received any CS training in their career and how often CS training was organized at their hospital in the last three years. Final questions included physicians’ willingness to participate in CS training and the perceived helpfulness of external experts in CS training. All continuous variables had to be completed using a 6-point Likert scale (see above). We have presented the English translation of the first three parts of the questionnaire in Additional file 1: Appendix 1.

The fourth section of the questionnaire was based on the Communication Skills Attitude Scale (CSAS) questionnaire [35], which measures attitudes towards CS learning. We used the Chinese version as translated earlier [36]. Participants evaluated their agreement with each item on a 5-point Likert scale from 1 (strongly disagree) to 5 (strongly agree). Two subscales were calculated, a positive attitude scale (PAS) and a negative attitude scale (NAS) as done before [37]. The PAS shows a maximum score of 70 and a minimum score of 14, whereas the NAS ranges between 60 and 12.

We evaluated the internal consistency of the questionnaire, calculating Cronbach’s α of the entire questionnaire, of CSAS separately and of each CSAS subscale. A coefficient of ≥ 0.70 was considered acceptable [38].

### Recruitment

A publicity enterprise (Wuhan CNweb Pioneer Ltd) in Wuhan, China implemented and hosted this survey. We chose a nationwide perspective and decided to conduct our study without any restriction of location or medical specialty, aimed at cross-representation of physicians and to depict a variety of experiences, ages, specializations and settings. The online link for the study was first circulated at the Tongji Medical College of the Huazhong University of Science and Technology in China’s Hubei province. Further participants were recruited via virtual and physical snowball sampling [39] through personal contact networks of participants and groups on WeChat – the most widely and frequently used mobile social media interface in China [40]. The first wave of two-thirds of the participants was recruited virtually, after that direct contacts were made to ask for spreading the link to further institutions. The participants were informed directly about the objectives and target groups of our study, so the link could be shared with other clinical institutions. As we started from central China, Wuhan, a major share of the participants came from the central south region (see Table 1). Our sampling approach followed a non-random convenience sampling strategy that has been often used in clinical research [41].

**Table 1:** Demographic characteristics of the sample including means, standard deviations, frequencies and percentages (*N* = 772)

**Characteristics**	**Mean, (SD)**
**Age (mean, SD)**	38.75 (7.84)
**Working years (mean, SD)**	14.20 (8.58)
**Degree**	**Participants, n (%)**
Junior college	113 (14.6)
Bachelor	473 (61.3)
Master	126 (16.3)
Doctorate	60 (7.8)
**Specialization**	
Operating physicians	272 (35.2)
Non-operating physicians	500 (64.8)
**Number of patients treated per week**	
Less than 50	179 (23.2)
50–100	281 (36.4)
101–200	99 (12.8)
More than 200	213 (27.6)
**Hospital level**	
Category 1 (Tertiary)	447 (61.8)
Category 2 (Others)	295 (38.2)
Secondary	209 (27.1)
Primary	65 (8.4)
Resident physicians	21 (2.7)
**Work place (Region)**	
Anhui	5 (0.6)
Beijing	2 (0.3)
Chongqing	3 (0.4)
Fujian	3 (0.4)
Gansu	4 (0.5)
Guangdong	15 (1.9)
Guanxi	23 (3)
Guizhou	13 (1.7)
Hebei	23 (3)
Henan	28 (3.6)
Hubei	442 (57.3)
Hunan	5 (0.6)
Inner Mongolia	11 (1.4)
Jiangsu	9 (1.2)
Jiangxi	5 (0.6)
Jilin	8 (1)
Liaoning	27 (3.5)
Shaanxi	9 (1.2)
Shandong	48 (6.2)
Shanghai	36 (4.7)
Shanxi	1 (0.1)
Sichuan	6 (0.8)
Tianjin	1 (0.1)
Xinjiang	13 (1.7)
Yunnan	3 (0.4)
Zhejiang	22 (2.8)
Unknown	7 (0.9)
**Work place (grouped)**	
North	23 (3.0)
Northeast	35 (4.5)
East	128 (16.6)
Central south	543 (70.3)
Southwest	32 (4.1)
Northwest	4 (0.5)
**Total**	**772**

Recruitment was carried out from July to October 2020.

### Data analysis

Overall 1080 completed questionnaires were received. A total of 146 questionnaires were excluded from data analysis because the participants were not medical physicians. Participants who showed an inconsistent answering pattern (i.e. always giving the same answer on contradicting questions, suggesting that they were not carefully reading the items) were excluded. Questionnaires completed in less than 303 s, which was below the 10th percentile, were also excluded, assuming a lack of credibility. The final dataset comprised *N* = 772 participants.

Medical specialties of the participants were summed up in a dichotomous variable as operating and non-operating physicians. Hospitals in China are categorized into three care levels with smaller primary-care hospitals in townships that provide basic services; secondary-care hospitals that are mostly situated in medium-sized cities; and large tertiary hospitals that provide specialized medical services [30, 42]. Due to considerably higher number of tertiary hospital physicians in our sample, we organized hospital levels into two categories: Category 1 was composed of only tertiary hospitals while Category 2 comprised secondary, primary-level hospitals and resident physicians.

After descriptive analysis of all participants (including means, SDs, and frequencies), Chi-Square analysis was used for differences between groups when variables were dichotomous, otherwise independent t-tests were performed. Spearman-Rho correlations were calculated for an explorative analysis of associations between continuous variables and reported as r_*S*_. A *p* value of < 0.05 was considered statistically significant.

Data were analyzed using SPSS (Version 26.0) [43].

## Results

### Participant characteristics

The sample consisted of *N* = 772 participants (response rate = 71.5%) with 451 female (58.4%) and 321 male (41.6%) physicians. The majority (61.3%) had a Chinese bachelor’s degree in medicine. Others had a junior college degree (14.6%), master’s degree (16.3%) or a doctorate (7.8%). The most prominent medical specializations were surgery (*n* = 225, 29.1%), internal medicine (*n* = 141, 18.3%) and pediatrics (*n* = 93, 12%). The sample was distributed in 336 cities of 26 different provinces and municipalities throughout mainland China. The Hubei province had the highest representation (*n* = 442, 57.3%). Table 1 summarizes the demographic data.

### Status of CS training

The prevalence of CS training provided by the hospitals varied widely. A total of 466/772 participants (60.4%) had received at least one CS training during their career, whereas 306/772 (39.6%) had never had a CS training. Physicians in tertiary-care hospitals had received a CS training in 299/477 (62.7%) cases, while 167/295 (56.6%) of the others had received one. A share of 63.2% (316/500) of the non-operating physicians had received CS training during their career whereas only 55.1% (150/272) of the operating physicians had been provided with CS training.

A further question asked for the organization of CS training during the past three years. Tertiary-care hospitals had organized CS trainings to physicians during the past three years at least once in 373/477 (65.9%), while 193/295 (34.1%) of other hospitals offered this. Group differences were significant (χ^2^ (4, *n* = 772) = 16.8, p* < 0.05). Non-operating physicians had been offered a CS training during the past three years at least once in 373/500 cases (74.6%), the operating group was provided in 193/272 cases (71.0%). This difference was not significant with χ^2^ (4, *n* = 772) = 4.78, *p* = 0.25. More details are presented in Table 2.

**Table 2:** Answers to the question "How many times communication training was organized at your clinic in the past three years?" (frequencies and percentages of rows or column respectively)

	**Category 1 (Tertiary)** **N (% rows)**	**Category 2 (Others)** **N (% rows)**	**Operating physicians** **N (% rows)**	**Non-Operating physicians** **N (% rows)**	**All** **N (% column)**
never	104 (50.5)	102 (49.5)	127 (61.7)	79 (38.3)	206 (26.7)
once	54 (60.0)	36 (40.0)	52 (57.8)	38 (42.2)	90 (11.7)
less than 3 times	160 (67.2)	78 (32.8)	156 (65.5)	82 (34.5)	238 (30.8)
more than 3 times	74 (67.3)	36 (32.7)	74 (67.3)	36 (32.7)	110 (14.2)
more than 5 times	85 (66.4)	43 (33.6)	91 (71.1)	37 (28.9)	128 (16.6)
**Total**	**477 (61.8)**	**295 (38.2)**	**500 (64.8)**	**272 (35.2)**	**772 (100.0)**

Physicians also rated how much they would like to participate in a CS training. They were highly motivated (mean = 5.21, SD = 1.18 [5.12, 5.29]), showing a broad interest in improving CS through specific training. Participants expressed in general a positive opinion about the teachability of CS (mean = 4.74, SD = 1.25 [4.66, 4.83]) and were open to have an external expert as teacher (mean = 4.98, SD = 1.26 [4.89, 5.07]). The participants strongly agreed that patient-physician communication was important for clinical work (mean = 5.85, SD = 0.47 [5.81, 5.88]) (see Table 4).

### Current aspects of patient-physician relationship

The participants rated the current situation related to patient-physician relationship in China as highly stressful (mean = 4.52, SD = 1.26 [4.43, 4.60]). A total of 22/772 (2.8%) rated the relationship as full of conflicts, almost half of the participants (374/772, 48.4%) judged the relationship to be based on distrust, 301/772 (39.0%) to be based on respect and 75/772 (9.7%) based on trust.

The physicians reported to have experienced verbal attacks in the past three years by a patient or his/her family never in 159/772 (20.6%), once a year in 372/772 cases (48.2%), once a month in 185/772 cases (24.0%), once a week/772 in 46 (6%) and every day in 10/772 cases (1.3%). Physical attacks were reported never in 655/772 (84.8%), once a year 111/772 times (14.4%), once a month in 5/772 cases (0.6%) and every day in 1/772 case (0.1%). Having received a previous CS training correlated negatively with the amount of verbal attacks with r_S_ = -0.07 (*p* = 0.06 [-0.14, 0.00]) and physical attacks with r_S_ = -0.09 (*p* = 0.01* [-0.16, -0.03]).

Answering to the multiple-choice question how patient-physician disputes had been resolved during the past three years they answered: by proactive communication with the patient in 256/771 (33.2%) cases, by communication through the department director in 131/771 (17%) cases, by communication through the hospital academy director in 168/771 (21.8%) cases, by means of mediation in 40/771 (5.2%) cases, and by a legal process in 44/771 (5.7%) cases (one person missing).

The participants assessed potential causing factors and the best way to resolve patient-physician disputes as presented in Table 3.

**Table 3:** Results to the questions "What are the factors causing the current situation of patient-physician relationship?" and “What do you think is the best way to resolve patient-physician disputes?" (means, standard deviations, and 95% confidence intervals)

	**mean, SD**	**95%- CI [LL, UL]**
**Causing factors for patient-physician disputes**		
Inadequate communication skills	4.54, 1.26	[4.46, 4.63]
Inadequate clinical skills	3.96, 1.44	[3.86, 4.08]
Lack of time	4.74, 1.30	[4.65, 4.83]
Low salaries/income	4.09, 1.52	[3.99, 4.20]
Doctors’ attitude	4.95, 1.22	[4.87, 5.04]
Patient’s behavior	5.28, 0.97	[5.21, 5.35]
Bureaucratic hurdles/barriers	5.05, 1.10	[4.97, 5.13]
New medical policy	5.38, 0.95	[5.31, 5.45]
**The best way to resolve patient-physician disputes**		
Improving communication skills	5.42, 0.94	[5.36, 5.49]
Improving clinical skills	5.36, 1.01	[5.29, 5.43]
Improve doctors time and process- management skills	5.33, 0.99	[5.26, 5.40]
Improve doctors’ income	5.31, 1.02	[5.24, 5.39]
Reduce proportion of patients' personal payments	4.99, 1.30	[4.91, 5.08]
Improving medical education	5.41, 0.95	[5.34, 5.47]
Improving hospital management	5.48, 0.89	[5.41, 5.54]

At the same time, physicians clearly recognized the positive effects of a good patient-physician relationship on clinical treatment. The results of the continuous variables are summarized in Table 4.

**Table 4:** Status of CS training, aspects of patient-physician relationship and perceived CS and patient-centeredness (means, standard deviations, and 95% confidence intervals)

Status of CS training	Mean, SD 95% CI [LL, UL]
Would you like to participate in a training of communication skills between physicians and patients?	5.21, 1.18 [5.13, 5.29]
Will it be helpful for you if external experts are invited to your institute for training?	4.98, 1.26 [4.89, 5.07]
Can patient-physician communication skills be taught via courses?	4.74, 1.25 [4.66, 4.83]
Do you think that patient-physician communication is important for clinical work?	5.85, 0.47 [5.81, 5.88]
Does good patient-physician relationship have a positive effect on clinical treatment?	5.70, 0.63 [5.66, 5.75]
**Current aspects of patient-physician relationship**	
Is the current patient-physician relationship stressful for you and your colleagues?	4.52, 1.26 [4.43, 4.60]
**Perceived CS and patient-centeredness**	
How do you rate your personal communication skills?	4.62, 0.92 [4.55, 4.68]
I inform my patients about advantages and disadvantages of a treatment plan	5.61, 0.72 [5.56, 5.66]
I take my patients’ opinion into account when choosing a treatment plan	5.69, 0.63 [5.64, 5.73]
I accept the decision of my patient event if it is not my favorite	4.85, 1.11 [4.77, 4.92]

### Perceived CS and patient-centeredness

The majority rated their own communication skills between somewhat good and good (mean = 4.62, SD = 0.92 [4.55, 4.68], showing confidence in their communication abilities. Self-assessed patient-centeredness was high in the aspects of informing patients (mean = 5.61, SD = 0.72 [5.56, 5.66]), taking the patients’ opinion into account (mean = 5.69, SD = 0.63 [5.64, 5.73]), and accepting an opposing decision of patients (mean = 4.85, SD = 1.11 [4.77, 4.92]).

### Attitudes towards CS training

The mean PAS was 62.96 (SD = 7.63 [62.41, 63.47]) indicating a high positive attitude towards CS training. Negative attitudes as measured by the NAS scores were low (mean = 26.72, SD = 8.38 [26.13, 27.30]). The results of the CSAS at the level of single items are presented in Additional file 2: Appendix 2.

### Predictors of verbal and physical attacks

Two binary regression models predicted the occurrence of having experienced verbal or physical attacks during the past three years: Being female was associated with an increased risk of verbal attacks while working in a tertiary hospital and showing high levels of PAS decreased this risk. Having received a previous training and high levels in PAS decreased, while higher levels in NAS increased the odds of physical attacks. For details see Table 5.

**Table 5:** Results of two binary regression models with regression coefficients (B), standard errors, odds ratios, and 95% confidence intervals

Variable	Verbal Attacks		Physical Attacks	
	B (SE)	OR^a^ [95% CI]	B (SE)	OR^a^ [95% CI]
Gender (female)	0.41 (0.20)	1.51^*^ [1.01, 2.25]	0.34 (0.22)	1.41 [0.91, 2.17]
Non-operating physician	-0.11 (0.21)	0.89 [0.60, 1.34]	0.33 (0.22)	1.40 [0.90, 2.16]
Tertiary hospital	-0.48 (0.19)	0.62^*^ [0.43, 0.89]	-0.28 (0.22)	0.76 [0.50, 1.16]
Previous training	-0.18 (0.19)	0.83 [0.58, 1.20]	-0.62 (0.22)	0.54* [0.35, 0.83]
PAS	-0.05 (0.01)	0.95^*^ [0.93, 0.98]	-0.03 (0.01)	0.97* [0.95, 1.00]
NAS	0.01 (0.01)	1.01 [0.99, 1.03]	0.03 (0.01)	1.03* [1.00, 1.05]

^a^Odds Ratio, ^*^p < .05

## Discussion

In this study, we provide a broad overview of the prevalence of CS training in China and the physicians’ attitudes towards and experiences with CS training and its possible implications on the current patient-physician relationship. Widespread reforms in the Chinese medical education and healthcare system in recent decades have impacted physicians and patients in unexpected ways. Stressful patient-physician relationships remain a burning issue, with broadening distrust and violence generating unsatisfied patients and burnout in physicians [11, 14, 44]. Amid educational and professional challenges, there is renewed interest in identifying competencies and requirements for being “a good physician” beyond the traditional clinical one [45, 46] with the role of communication and CS training gaining value [47, 48].

### Perspectives on the status of CS training

Our results confirmed that medical institutions across mainland China still do not prioritize providing CS training to physicians. Almost 40% of our participants reported not receiving any CS training at all during their careers and over one-fourth of the physicians reported that no training was organized within the past three years at their respective hospitals. While the number of CS trainings offered by institutions was still low, tertiary hospitals had organized more CS training courses in the past three years when compared to secondary and primary hospitals, which is in accordance with recent findings [49]. Apparently, the primary healthcare system is suffering from lower educated staff, lower care-quality, and lower reimbursement rates [16].

Public hospitals in China are designated into primary, secondary and tertiary categories based on the hospital size and availability of medical services at the institution. Since tertiary hospitals are large and sufficiently funded to provide comprehensive specialist health services and medical education and to conduct scientific research [50], the differences in our results could be explained with the higher need for adequately trained physicians at tertiary hospitals because of larger number of patients and a higher potential for patient-physician disputes. Despite the low prevalence of CS training, our results suggest that tertiary hospitals have realized the benefits of CS training for physicians and are better placed to implement such courses. A recent study in 47 public tertiary hospitals in Shanghai reported that physicians widely utilized patient-centered communication, such as shared decision-making concepts, which had improved patient care and satisfaction [34]. However, as our survey did not specifically inquire which aspects of CS training were provided to physicians, for instance role-play or interaction with standardized patients, the details of the formats or content of these training courses could not be determined with our results.

In our sample, physicians working in non-operating fields had slightly higher attendance rates for CS training than operating physicians (e.g. surgeons and dentists). This finding could be explained by the personal need for better communication skills felt by non-operating physicians as opposed to operating physicians, whose interaction with patients is usually less communicative. However, the consequences for patients and operating physicians arising out of post-operative complications emphasize the need for CS training for all physicians. Empirical studies comparing communication skills of surgeons or communicative demands in a surgical consultation compared with other specialties are rare. Findings from a Canadian study showed that surgeons were performing less "information giving" than general practitioners [51]. However, comparisons with Chinese physicians might be questionable and more research is needed on the communicative needs within different specialties in China.

### Challenging aspects in the patient-physician relationship and the role of CS attitudes

Our participants rated the overall situation of patient-physician relationships in China as highly stressful and based on distrust, as reported previously [14]. A significant share of our sample reported to already have experienced verbal or physical attacks within the past three years. However, verbal attacks were more prevalent. An important finding of our study is that those with experiences in former CS trainings reported significantly fewer physical attacks.

Causing factors of patient-physician disputes in our sample were rooted predominantly in medical policies, patient's behavior, and bureaucratic barriers by the participants of our study. In fact, the new medical policy that has been initiated by the improvements due to health care reforms, reached the highest value as a potential causing factor of disputes. This may be due to high expectations of patients regarding the new role of physicians, who in turn were not properly prepared to fulfill these expectations. Our results confirm this assumptions, as the best ways to resolve these disputes were seen by all participants in improving CS, education and hospital management. In line with this, the physicians' general attitude towards receiving CS training was positive, as shown by high scores on the PAS accompanied with the strong belief that communication skills could be taught. As expected by such positive mindset, the physicians showed a high willingness to improve their CS through courses.

However, these overall positive results may be partially due to floor or ceiling effects of our new questionnaire, as more than 15% of the participants reached the highest or in case of the NAS the lowest possible scores [52]. All participants seemed to answer in accordance with nearly all causes of patient-physician disputes except in case of clinical skills and physicians' income. Moreover, nearly all participants rated the status of CS training and perceived own CS and patient-centeredness homogeneously, as well as the PAS. Interestingly, the self-assessment of stress, the rating of personal communication skills, and some aspects of the NAS were not affected by ceiling and floor effects. Future instruments can be developed based on these results to provide measurements with a higher discriminatory power.

Nonetheless, a general positive mentality, as specifically shown in our regression model, might help to reduce the occurrence of verbal and physical attacks. Verbal attacks seemed to have a higher prevalence towards female physicians, which hints at a potential pathway to targeted and gender-specific CS trainings. Additionally, having received previous trainings significantly helped to avoid physical attacks. This association strongly suggests a mediating role of communication and a possible helpful approach to avoid or solve conflicts and rebuild patient-physician trust through better communication. While court documents show dissatisfaction with treatment processes and outcomes to be the most prevalent reasons for serious violent attacks [53], physicians blame economic pressure, a highly asymmetric physician–patient relationship, and low levels of training in communication and humanities for this situation [54]. There exists a strong need not only for CS training but as well for de-escalation and inter-collegial support. Moreover, trainings should also include skills on how to properly differentiate between patients' aggression caused by understandable frustration and premeditated intentions.

Last not least, although the health insurance covers main medical services in China, there is still a high financial burden for the poor population out of which more than 40% is impoverished due to illness [55]. Especially agricultural migrants that cover one fifth of the Chinese population suffer from the low reimbursement rates of the insurance schemes [56] which show an annual reimbursement ceiling of a six time average wage of a local farmer for rural residents [2]. In-depth interviews with patients from different professional groups are necessary to understand the real reasons for the tense relationship they feel towards the medical system in China and their representatives.

### Role of culture in CS training

Current Western standards of training communicative competencies to physicians require high levels of professionalism that have been defined in frameworks and integrated into the medical curricula [57]. Good patient-physician communication from a western perspective includes showing interest in patients' opinions and feelings, and disclosing full information in order to come to a shared decision for a treatment [58]. This expectation towards adequate communication reflects a deep change in understanding, which is quite recent. Up to the 1950s, sociologists such as Talcott Parsons described doctor-patient relationships as a highly asymmetric and paternalistic way of interaction [59]. A rather asymmetrical doctor-patient relationship is not only prevalent in China, but generally in Asia [60], where the role of the doctor is driven by economic pressures on the one hand and highly hierarchical organizational structures on the other hand. Therefore, CS training has to meet the needs of these doctors who cannot simply adopt Western models in their practice. For instance, communication with family members plays a significant role in patient-physician relationship [61], but a substantial share of the population in this region is less educated and fully informing them is challenging for doctors who are neither trained on communicating nor paid for adequately and finally might feel restrictions by their respective institutions.

### Implications for Chinese medical education

Our results confirm the recommendations of previous studies to invest further efforts in the Chinese healthcare system and in medical education [45]. Dedicated efforts are needed to increase the attractiveness of the medical profession for young people and to help establish a better reputation of the profession by reducing patient-physician conflicts, especially in the primary health-care system [16] but also in a long-term national strategy [18]. Moreover, medical humanities such as medical psychology, sociology, and ethics have been identified as necessary parts of a teaching curriculum to teach professionalism. Chinese doctors report a deep lack of education in this respect [54] and although medical colleges started to implement social courses they still lag far behind Western models [62]. A special focus on teaching CS with various didactical methods, including simulation patients, will be beneficial [63].

### Limitations and future directions

Although our sample was spread all over mainland China, a majority of participants were from the Hubei province (57.3%), a relatively smaller region in Central China with a population of 57 million people. Our sample may not be truly representative since there are more than 3.4 million registered physicians in China, according to the statistical yearbook 2021 [64]. The majority of our participants (85.6%) held at least a bachelor’s degree which shows a higher level of education than reported earlier for common resident physicians [19]. Another important aspect is that our survey focused solely on the physicians’ attitudes and experience with CS, while the effects on patients or their experiences were not investigated. Now that a general positive attitude for physicians can be confirmed, future research should focus on which types and formats of training are common practice, which are missing, and which ones give better results (for patients and physicians), so that future investments can be better directed.

## Conclusions

Effective communication is a core competence for physicians to properly communicate with their patients. Considering that the Chinese healthcare system has rapidly improved in the previous decade, a considerable gap exists in training physicians on developing adequate communication skills in order to reduce conflicts and improve the patient-physician relationship. This study is unique in that it reports on the status of CS training and the physicians’ experiences and attitudes towards CS training from all over China.

We found that about 60% of the hospitals had organized CS training at least once in the past three years and tertiary hospitals had organized a higher number of trainings as compared to primary and secondary hospitals. Risk factors for the occurrence of verbal and physical attacks could be detected as follows: Being female, working at a primary or secondary hospital, and lower positive attitude towards CS were determined as risk factors for verbal attacks. High levels of positive attitudes of CS and having received a previous CS training, as well as low levels of negative attitudes of CS increased the risk of physical attacks. CS training was viewed positively by the physicians as a tool to improve patient-physician relations and clinical treatment outcomes. Physicians were welcoming towards more offers for CS courses, and their positive attitudes correlated with the use of patient-centered communication in practice. Given the highly stressful patient-physician relationship in China, more efforts and investments are essential to provide CS training to physicians at hospitals, especially on primary and secondary levels.

## Supplementary Information

**Additional file 1: Appendix I.** Communication Skills Questionnaire.

**Additional file 2: Appendix II.** Results of the Communication Skills Attitudes Scale: Items, direction of scale, means, standard deviations, and 95% confidence intervals (*N* = 772).

## Data Availability

The datasets used and/or analyzed during the current study are available from the corresponding author on reasonable request.
