# Supplementary material for: Communication Skills (CS) training of physicians in China and its role in actual challenges of patient-physician relationship: a cross-sectional survey
Source: BMC Med Educ. 2022 Nov 12;22:783. doi: 10.1186/s12909-022-03830-9 (PMC9652837; doi:10.1186/s12909-022-03830-9)
Supplement: Supplementary file 1 — Additional file 1: Appendix I. Communication Skills Questionnaire. [file 12909_2022_3830_MOESM1_ESM.docx]

Appendix I: Communication Skills Questionnaire

# Part I: Demographic details

| What is your gender? | □ male □ female | |
| --- | --- | --- |
| What is the month/year of your birth? | ______________ year _________________ month | |
| What is your level of medical education? | □ junior college □ bachelor’s  □ master’s □ doctorate | |
| Which city do you currently work in? | _______ ________ | |
| Which hospital are you currently working at? |  | |
| What is the level of your hospital? | □ high level □ middle level  □ basic level □ practical | |
| What is your specialization? | □ internal medicine  □ surgery  □ plastic & aesthetics  □ paediatrics  □ ophthalmology  □ gynaecology & obstetrics | □ ENT  □ dentist  □ anaesthesia  □ emergency medicine  □ psychiatry  □ other: ­­­­­­­­­­­­­­­­­­­­___________ |
| How many years have you worked as a doctor? | ___________________________ years | |
| How many patients do you treat per week (including outpatient and in-patient)? | □ <50 □ 50-100 □ 101-200 □ > 200 | |
| How do you rate your personal communication skills? | \| 1 \| 2 \| 3 \| 4 \| 5 \| 6 \| \| --- \| --- \| --- \| --- \| --- \| --- \| \| Very bad \| Bad \| Somewhat bad \| Somewhat good \| Good \| Very good \| | |

# Part II: Doctor-patient relationship

By choosing the most appropriate response on the given scale of 1 to 6, please indicate the degree of agreement with the following questions and statements.

| **Strongly disagree** | **Disagree** | **Slightly disagree** | **Slightly agree** | **Agree** | **Strongly agree** |
| --- | --- | --- | --- | --- | --- |
| **1** | **2** | **3** | **4** | **5** | **6** |
| **Worst** | **Worse** | **Bad** | **Good** | **Better** | **Best** |

| Can doctor-patient communication skills be taught via courses? | | | 1 | | 2 | | 3 | | 4 | | 5 | 6 | |
| --- | --- | --- | --- | --- | --- | --- | --- | --- | --- | --- | --- | --- | --- |
| How would you rate the current doctor-patient relationship in China in general? Is it   - full of conflicts - based on distrust - based on respect - based on trust | | | | | | | | | | | | | |
| Is the current doctor-patient relationship stressful for you and your colleagues? | | | 1 | | | 2 | | 3 | | 4 | 5 | | 6 |
| In the past three years, have you experienced a verbal attack by a patient or his/her family?   - never - once a year - once a month - once a week - every day | | | | | | | | | | | | | |
| In the past three years, have you experienced a physical attack by a patient  or his/her family?   - never - once a year - once a month - once a week - every day | | | | | | | | | | | | | |
| At what level of communication have you resolved your doctor-patient disputes in the past three years? | | | | | | | | | | | | | |
| a) Proactive communication with the patient | | 1 | | | 2 | | 3 | | 4 | | 5 | 6 | |
| b) Communication through the department director | | 1 | | | 2 | | 3 | | 4 | | 5 | 6 | |
| c) Communication through the hospital academy director | | 1 | | | 2 | | 3 | | 4 | | 5 | 6 | |
| d) Mediation | | 1 | | | 2 | | 3 | | 4 | | 5 | 6 | |
| e) Through the legal process | | 1 | | | 2 | | 3 | | 4 | | 5 | 6 | |
| What do you think is the best way to resolve doctor-patient disputes? | | | | | | | | | | | | | |
| a) Improving communication skills | 1 | | | 2 | | | 3 | | 4 | | 5 | 6 | |
| b) Improving clinical skills | 1 | | | 2 | | | 3 | | 4 | | 5 | 6 | |
| c) Improve doctors time and process- management skills | 1 | | | 2 | | | 3 | | 4 | | 5 | 6 | |
| d) Improve doctors’ income | 1 | | | 2 | | | 3 | | 4 | | 5 | 6 | |
| e) Reduce proportion of patients' personal payments | 1 | | | 2 | | | 3 | | 4 | | 5 | 6 | |
| f) Improving medical education | 1 | | | 2 | | | 3 | | 4 | | 5 | 6 | |
| e) Improving hospital management | 1 | | | 2 | | | 3 | | 4 | | 5 | 6 | |
| What are the factors causing the current situation of doctor-patient relationship? | | | | | | | | | | | | | |
| a) Inadequate communication skills | | 1 | | | 2 | | 3 | | 4 | | 5 | 6 | |
| b) Inadequate clinical skills | | 1 | | | 2 | | 3 | | 4 | | 5 | 6 | |
| c) Lack of time | | 1 | | | 2 | | 3 | | 4 | | 5 | 6 | |
| d) Low salaries/income | | 1 | | | 2 | | 3 | | 4 | | 5 | 6 | |
| e) Doctors’ attitude | | 1 | | | 2 | | 3 | | 4 | | 5 | 6 | |
| f) Patient’s behaviour | | 1 | | | 2 | | 3 | | 4 | | 5 | 6 | |
| g) Bureaucratic hurdles/barriers | | 1 | | | 2 | | 3 | | 4 | | 5 | 6 | |
| h) New medical policy | | 1 | | | 2 | | 3 | | 4 | | 5 | 6 | |
| A good doctor-patient relationship has a positive effect on clinical treatment (outcome) | | 1 | | | 2 | | 3 | | 4 | | 5 | 6 | |
| I inform my patients about the advantages and disadvantages of a treatment plan | | 1 | | | 2 | | 3 | | 4 | | 5 | 6 | |
| I take my patients’ opinion into account when choosing a treatment plan | | 1 | | | 2 | | 3 | | 4 | | 5 | 6 | |
| I accept the decision of my patient even if it is not my favourite | | 1 | | | 2 | | 3 | | 4 | | 5 | 6 | |

**Part III: Teaching of Doctor-patient communication skills**

| Can doctor-patient communication skills be taught via courses? | 1 | 2 | 3 | 4 | 5 | 6 |
| --- | --- | --- | --- | --- | --- | --- |
| Have you received any communication skills training previously?   - Yes - No | | | | | | |
| How many times communication training was organised at your clinic in the past 3 years?   - More than 5 times - More than 3 times - Less than 3 times - Once in 3 years - Never | | | | | | |
| Would you like to participate in a training of communication skills between doctors and patients? | 1 | 2 | 3 | 4 | 5 | 6 |
| Will it be helpful for you if external experts are invited to your institute for training? | 1 | 2 | 3 | 4 | 5 | 6 |
