# Supplementary material for: Communication Skills (CS) training of physicians in China and its role in actual challenges of patient-physician relationship: a cross-sectional survey
Source: BMC Med Educ. 2022 Nov 12;22:783. doi: 10.1186/s12909-022-03830-9 (PMC9652837; doi:10.1186/s12909-022-03830-9)
Supplement: Supplementary file 2 — Additional file 2: Appendix II. Results of the Communication Skills Attitudes Scale: Items, direction of scale, means, standard deviations, and 95% confidence intervals (N = 772). [file 12909_2022_3830_MOESM2_ESM.docx]

**Appendix II: Results of the Communication Skills Attitudes Scale: Items, direction of scale, means, standard deviations, and 95% confidence intervals (N = 772)**

| **Nr.** | **Item** | **PAS/NAS*** | **M** | **SD** | **95 %- CI [LL, UL]^a^** |
| --- | --- | --- | --- | --- | --- |
| 1. | In order to be a good doctor I must have good communication skills | PAS | 4.77 | 0.51 | [4.74, 4.81] |
| 2. | I can't see the point in learning communication skills | NAS | 1.72 | 1.19 | [1.63, 1.80] |
| 3. | Nobody is going to fail their medical degree for having poor communication skills | NAS | 3.15 | 1.48 | [3.05, 3.27] |
| 4. | Developing my communication skills is just as important as developing my knowledge of medicine | PAS | 4.67 | 0.66 | [4.62, 4.72] |
| 5. | Learning communication skills has helped or will help me respect patients | PAS | 4.61 | 0.74 | [4.56, 4.66] |
| 6. | I haven't got time to learn communication skills | NAS | 2.46 | 1.33 | [2.37, 2.55] |
| 7. | Learning communication skills is interesting | PAS | 4.35 | 0.88 | [4.29, 4.41] |
| 8. | I can't be bothered to turn up to sessions on communication skills | NAS | 1.78 | 1.11 | [1.70, 1.86] |
| 9. | Learning communication skills has helped or will help facilitate my team-working skills | PAS | 4.65 | 0.63 | [4.60, 4.69] |
| 10. | Learning communication skills has improved my ability to communicate with patients | PAS | 4.66 | 0.62 | [4.61, 4.70] |
| 11. | Communication skills teaching states the obvious and then complicates it | NAS | 2.31 | 1.46 | [2.21, 2.41] |
| 12. | Learning communication skills is fun | PAS | 4.23 | 0.92 | [4.16, 4.30] |
| 13. | Learning communication skills is too easy | NAS | 2.86 | 1.14 | [2.79, 2.93] |
| 14. | Learning communication skills has helped or will help me respect my colleagues | PAS | 4.39 | 0.81 | [4.33, 4.45] |
| 15. | I find it difficult to trust information about communication skills given to me by non-clinical lecturers | NAS | 2.88 | 1.41 | [2.79, 2.98] |
| 16. | Learning communication skills has helped or will help me recognise patients' rights regarding confidentiality and informed consent | PAS | 4.31 | 0.93 | [4.24, 4.37] |
| 17. | Communication skills teaching would have a better image if it sounded more like a science subject | PAS | 4.26 | 0.91 | [4.19, 4.32] |
| 18. | When applying for medicine, I thought it was a really good idea to learn communication skills | PAS | 4.51 | 0.72 | [4.46, 4.56] |
| 19. | I don't need good communication skills to be a doctor | NAS | 1.58 | 1.17 | [1.50, 1.66] |
| 20. | I find it hard to admit to having some problems with my communication skills | NAS | 2.64 | 1.26 | [2.55, 2.72] |
| 21. | I think it's really useful learning communication skills on the medical degree | PAS | 4.56 | 0.74 | [4.51, 4.60] |
| 22. | My ability to pass exams will get me through medical school rather than my ability to communicate | NAS | 3.60 | 1.32 | [3.51, 3.69] |
| 23. | Learning communication skills is applicable to learning medicine | PAS | 4.37 | 0.86 | [4.31, 4.43] |
| 24. | I find it difficult to take communication skills learning seriously | NAS | 2.35 | 1.38 | [2.24, 2.45] |
| 25. | Learning communication skills is important because my ability to communicate is a lifelong skill | PAS | 4.63 | 0.67 | [4.59, 4.68] |
| 26. | Communication skills learning should be left to psychology students, not medical students | NAS | 2.04 | 1.44 | [1.94, 2.15] |

^a^ Unless otherwise noted, bootstrap results are based on 1000 bootstrap samples

^*PAS/NAS: Positive Attitudes Scale/ Negative Attitude Scale^
